# Intravenous infusion of H_2_-saline suppresses oxidative stress and elevates antioxidant potential in Thoroughbred horses after racing exercise

**DOI:** 10.1038/srep15514

**Published:** 2015-10-23

**Authors:** Masahiko Yamazaki, Kanichi Kusano, Toru Ishibashi, Masataka Kiuchi, Katsuhiro Koyama

**Affiliations:** 1 Interdisciplinary Graduate School of Medicine and Engineering, University of Yamanashi, 4-4-37 Takeda, Kofu, Yamanashi 400-8510, Japan; 2 Yamazaki Horse Clinic, 2-1-1 Mikoma, Miho-mura, Inasiki-gun, Ibaraki 300-0415, Japan; 3 Japan Racing Association, Deputy Manager, Equine Department Veterinary Section, 11-1, Roppongi 6-chome Minato-ku, Tokyo 106-8401, Japan; 4 Department of Rheumatology and Orthopaedic Surgery, Huis Ten Bosch Satellite H2 Clinic Hakata, 2-1 Gion, Hakata-ku, Fukuoka 812-0038, Japan; 5 Anicom Speciality Medical Institute, 1-5-22 Shimoochiai, Shinjuku-ku, Tokyo 161-0033, Japan; 6 Graduate School Department of Interdisciplinary Research, University of Yamanashi 4-4-37 Takeda, Kofu, Yamanashi 400-8510, Japan

## Abstract

Upon intensive, exhaustive exercise, exercise-induced reactive oxygen species may exceed the antioxidant defence threshold, consequently resulting in muscular damage or late-onset chronic inflammation. Recently, the therapeutic antioxidant and anti-inflammatory effects of molecular hydrogen (H_2_) for human rheumatoid arthritis have been demonstrated. However, it is also important to clarify the effects of administrating H_2_ in large animals other than humans, as H_2_ is thought to reach the target organ by passive diffusion upon delivery from the blood flow, indicating that the distance from the administration point to the target is critical. However, data on the effects of H_2_ on oxidative stress in real-life exhaustive exercise in large animals are currently lacking. We here investigated 13 Thoroughbred horses administered intravenous 2-L saline with or without 0.6-ppm H_2_ (placebo, N = 6; H_2_, N = 7) before participating in a high-intensity simulation race. Intravenous H_2_-saline significantly suppressed oxidative stress immediately, 3 h, and 24 h after the race, although the antioxidant capability was not affected throughout the study. The serum creatine kinase, lactate, and uric acid levels were increased in both groups. Taken together, these results indicate that intravenous H_2_-saline can significantly and specifically suppress oxidative stress induced after exhaustive racing in Thoroughbred horses.

Muscular damage or late-onset chronic inflammation can result from exercise-induced reactive oxygen species (ROS). The balance between these exercise-induced ROS and the defence mechanisms by intrinsic or extrinsic antioxidants plays an important role in maintaining energy homeostasis and mitochondrial functions. These effects of exercise-induced ROS are largely regulated by the peroxisome proliferator-activated receptor-γ co-activator 1α (PGC-1α)-related pathways, including AMP-activated protein kinase and sirtuin 1, in combination with various energy metabolic products^1^,^2^. PGC-1α directs the exercise-related mitochondrial biogenesis, resulting in increase in oxygen supply to the muscle^3^ and over-generation of ROS accompanied by electron leakage from the mitochondrial respiratory chain^4^. Despite the increase in metabolic sources for ROS during intensive and exhaustive exercises, the deleterious effects by ROS are suppressed by the adaptive induction of numerous intrinsic ROS-elimination mechanisms, including superoxide dismutase, glutathione peroxidase, and non-enzymatic glutathione^5^. Endurance exercise can stimulate the release of anti-inflammatory cytokines^6^,^7^, and, therefore, it is considered to prevent the morbidity of age-related chronic inflammatory diseases such as diabetes, cancer, atherosclerosis, and cardiovascular disorders.

In contrast to endurance training, intensive exercise, including for example human athletics and horse races, requires fast and high-power contraction of white muscles rich in fast type IIx fibres, in which the majority of energy is supplied through glycolytic and anaerobic reactions^8^. Although the role of ROS in anaerobic exercise, in which the energy supply is quickly exhausted, is not fully understood, the majority of such anaerobic strenuous exercises continue over 30 seconds up to several minutes, thereby subsequently or simultaneously requiring aerobic energy metabolism^9^,^10^. Under such circumstances, oxidative stress is thought to largely regulate the muscle conditions after exercise^11^,^12^,^13^, and it is thus important to balance the oxidative state with that of the antioxidant defence capabilities.

On the other hand, during such exhaustive exercises, the threshold for maintaining architectural or stoichiometric strength of the skeletal muscle is frequently exceeded, resulting in muscle damage, accompanied by elevation of various serum biomarkers, including creatine kinase (CK), aspartate aminotransferase (AST), alanine aminotransferase, and lactate dehydrogenase (LDH), which are released through the affected muscle cell membrane^14^,^15^,^16^. It has been hypothesised that chemical stress, as represented by ROS, during strenuous exercise increases the permeability of muscle cells by peroxidation of the cell membranes^17^,^18^, thereby promoting leakage of the intracellular enzymes^19^. Furthermore, the rapid depletion of anaerobic energy sources such as free ATP, phosphocreatine, and glycogen results in the degradation of AMP to generate inosine 5′-monophosphate and ammonia^20^,^21^; therefore, the final metabolite of purine nucleotide metabolism, uric acid (UA), is a useful marker for the exhaustion of energy sources in addition to serum lactate, which also increases upon depletion of the glycolytic energy supply^22^,^23^,^24^.

These deleterious influences by exercise are not restricted to strenuous exercise. For example, in elderly people^25^ and individuals with sarcopoenia^26^,^27^ or chronic inflammatory diseases^28^, non-intensive exercise can easily exceed the resistance limitations of the muscles. In such conditions, muscle atrophy is common, thereby impairing the antioxidant defence capabilities, as well as the anti-inflammatory systems. Therefore, it is important to determine methods to prevent the excess ROS from overwhelming the antioxidant defence potential; this would not only be useful for protecting skeletal muscle from injury, which is hard to avoid in high-intensity exercises such as horse racing, as well as in human athletic exercises, but also for successfully designing rehabilitation strategies for morbid or wasted muscles.

Among numerous antioxidants that are exogenously absorbable, molecular hydrogen (H_2_) has shown promising anti-inflammatory potential, as well as high bio-safety^29^,^30^,^31^. In addition to its anti-inflammatory properties, H_2_ also appears to show protective effects on the muscle against the detrimental complications accompanied by excessive exercise-related oxidative stress. Recently, the reducing potential of H_2_ against oxidative stress in atrophic muscle in rodents^32^ and a possibility that H_2_ may work as an antioxidant during treadmill exercise in Thoroughbred horses^33^ have been reported. However, direct evidence regarding whether H_2_ possesses antioxidant properties or protective effects for skeletal muscle exposed to strenuous exercise is still lacking.

Further, it should be noted that the body size of the experimental model used is also important. Because H_2_ is thought to distribute throughout the body by passive diffusion upon delivery from the blood flow, the H_2_ molecules that reach the target organ is in inverse proportion to the cube of the distance from the area of administration. Most of the investigations concerning H_2_ therapy are performed using small animals such as rodents, and there is a large discrepancy of the distances from the administration point to the target organ between rodents and humans and other large mammals. To assess the accessibility of H_2_, we tested the efficacy of intravenous infusion of approximately 0.6-ppm H_2_ dissolved saline (H_2_-saline) in thoroughbred horses exposed to extremely high levels of oxidative stress by high-intensity competitive races. Further, to clarify the protective and/or preventable capability of H_2_ against oxidative stress or muscle damages caused by high-intensity exercise, we investigated the oxidative state and several metabolic/injury biomarkers before and after simulation races in which placebo-controlled intravenous infusion of H_2_-saline was administered. To evaluate the oxidative or reducing potential present in the sera of the racing horses, we performed the diacron-Reactive Oxygen Metabolite (d-ROM) and Biological Antioxidant Potential (BAP) tests, respectively^34^,^35^. Moreover, among the biomarkers for oxidative stress, we particularly focused on the alteration of serum 8-hydroxydeoxyguanosine (8-OHdG), which directly reflects the consequences of oxidative stress, as well as the generation of mutagenic damages on DNA, which in turn may induce secondary, chronic morbidity.

## Results

### Analysis of oxidative stress and antioxidant potentials in the serum

**Figure 1:**
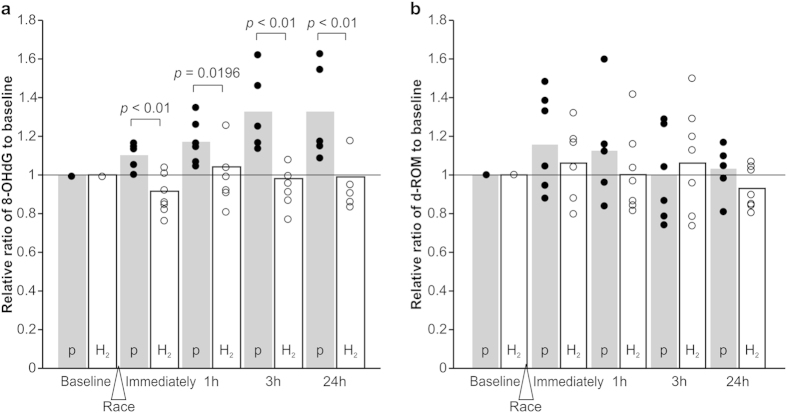
Variations in the relative ratios to baseline of (**a**) serum 8-hydroxydeoxyguanosine (8-OHdG) and (**b**) serum diacron-Reactive Oxygen Metabolites (d-ROM) immediately, and 1, 3, and 24 h after the race. The scatter plots indicate the value for each individual horse (closed circles: placebo [P] group, open circles: H_2_ group). The mean values of the relative ratios to baseline of serum 8-OHdG and d-ROM are shown as grey columns for the placebo group and white columns for the H_2_ group.

At baseline, the serum 8-OHdG levels were 1.29 ± 0.104 ng/mL in the placebo group and 1.45 ± 0.077 ng/mL in the H_2_ group. The relative ratios of 8-OHdG at each time point after the race to the baseline are presented in Fig. 1a. The 8-OHdG levels in the H_2_ group were significantly suppressed immediately, 3 h, and 24 h after the race compared to in the placebo group. On the other hand, the mean values of the d-ROMs, which is an indirect method to reflect the free radicals in the serum, were 149 ± 24.6 U.CARR in the placebo group and 154 ± 24.8 U.CARR in the H_2_ group at baseline, and no significant increases were observed during the study in either group (Fig. 1b).

**Figure 2:**
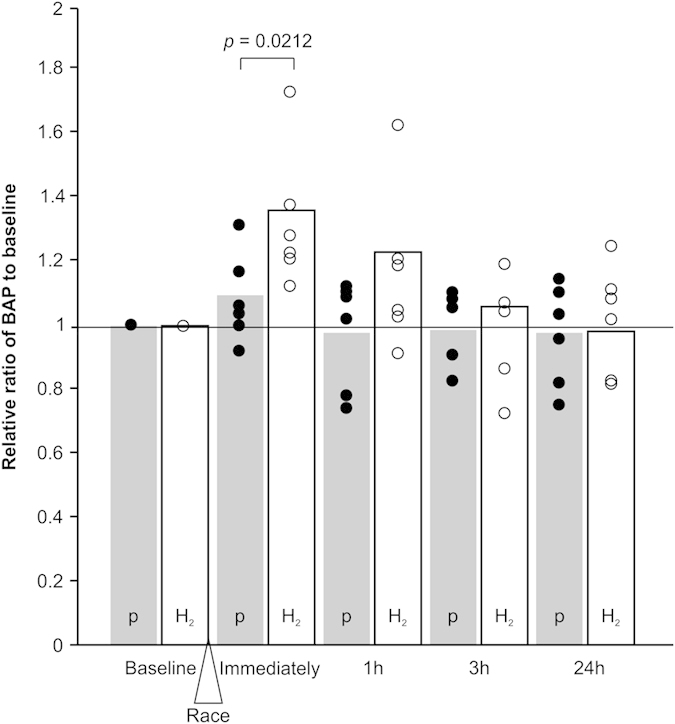
Variations in the relative ratio to baseline of the Biological Antioxidant Potential (BAP) scores in the serum immediately, and 1, 3, and 24 h after the race. The scatter plots indicate the value for each individual horse (closed circles: placebo [P] group, open circles: H_2_ group). The mean values of the relative ratios to baseline are shown as grey columns for the placebo group and white columns for the H_2_ group.

The BAP test was also performed to measure the reducing potential in the serum. The mean values of the BAP test, which reflects the reduction potential in the serum, were 3850 ± 251 μmol/L in the placebo group and 3560 ± 1100 μmol/L in the H_2_ group at baseline. The relative ratios of the scores at each time point after the race to the baseline are shown in Fig. 2. Although there was no significant difference between the H_2_ and placebo groups, the ratio tended to be elevated in the H_2_ group (36.8 ± 20.6%), as compared to in the placebo group (8.50 ± 12.6%; *p* = 0.0212) immediately after the race.

### Biomarkers for muscular damage and anaerobic metabolism during the race

As biomarkers for exercise-induced muscle damages, CK, AST, and LDH were measured (Table 1). In both groups, the CK levels were significantly increased 3 h after the race, but were restored to near the baseline values within 24 h after the race. There were no significant differences between the placebo and H_2_ groups. In both groups, the AST and LDH levels did not significantly change during the 24-h period after the race.

Next, to estimate the alterations induced by anaerobic metabolism, the serum lactate and UA levels were measured (Table 2). The serum lactate levels were significantly increased in both groups 1 h after the race. They reached their maximum levels (28.8 and 38.1-fold increases in the placebo and H_2_ groups, respectively) immediately after the race and reduced rapidly within 3 h, remaining 1.7 and 1.8-fold elevated compared to the baseline in the placebo and H_2_ groups, respectively, after 24 h. However, there were no significant differences in the lactate levels between the groups at any time point. On the other hand, the UA level was significantly increased immediately after the race only in the H_2_ group; at 1 h after the race, significant increases in the UA levels were observed in both groups. However, there were no significant differences between the groups. At 3 h after the race, the UA levels gradually decreased and were restored to near the baseline values within 24 h after the race.

## Discussion

In this study, intravenous infusion of H_2_-saline showed significant antioxidant effects in Thoroughbred horses after high-intensity racing exercise, as demonstrated by the prevention of increases in the formation of 8-OHdG in the H_2_ group compared to in the placebo group. This result strongly indicates protective effects of H_2_ against exercise-induced ROS-mediated detrimental tissue damage in racing horses. However, it should be noted that another assay to measure oxidative stress, namely d-ROM, showed no significant differences between the two groups. We speculate that this discrepancy between the 8-OHdG and d-ROM assays is likely caused by the indirect and non-specific metabolites detected by d-ROMs. For example, superoxide and H_2_O_2_, two major ROS, are balanced by the corresponding scavengers, superoxide dismutase and catalase, respectively. The oxidative stress measured by d-ROM may reflect that of the ROS after they are reduced by their specific scavengers. On the other hand, serum 8-OHdG is a direct and reliable marker for elevated oxidative stress, as it reflects oxidised DNA. Further, while 8-OHdG reflects the intracellular oxidation or oxidants that reach the DNA, d-ROM may reflect the extracellular oxidants, which may be scavenged in the serum and may not reach the intracellular components, such as the mitochondrial or genomic DNA. Therefore, we believe that the results obtained by serum 8-OHdG are reliable and accurate.

On the other hand, although a tendency of an elevation of the antioxidant potential in the serum measured by the BAP test was observed immediately after the race in the H_2_-saline infused group, no significant differences in the elevation of antioxidant potential was observed in either group throughout the study. Recently, it was demonstrated that, in humans in rest position, intravenously infused H_2_ emerged relatively slowly from the skin, and that it took more than 30 min after administration for it to be excreted^36^. In the present study, although there is a possibility that the H_2_ molecules administered 2 h before the race may have been partially retained in the body of the horses during and after the race, the H_2_ would have been discharged or consumed early during the race due to the high intensity of horse races, and therefore, elevation of the BAP value may not have been observed throughout the race except for immediately after the race. Alternatively, the discrepancy between the BAP and 8-OHdG values seems to suggest that the serum BAP value does not reflect the intracellular antioxidant potential, unlike 8-OHdG. These results suggest that the intravenously administered H_2_ molecules have reached the muscles cells and sustained the anti-oxidant potential even after the race. However, there remain the possibilities that the H_2_ molecules in blood were insufficient to reach the muscle cells for the racing exercise and instead, other unknown mechanisms which had worked on the cells or molecules in the circulating blood, may have indirectly suppressed 8-OHdG in the muscle cells.

There are numerous previous studies regarding muscular damage due to anaerobic exercise^37^,^38^,^39^. In the present study, although the AST and LDH levels did not significantly change in either group, the CK level was elevated equally in both groups upon racing, indicating the occurrence of muscular damage during horse racing (Table 1). Additionally, these results also indicate that infused H_2_ did not completely protect the muscular cells from damage caused by the exhaustive racing exercise. In addition, neutrophils, which may infiltrate into the muscle tissue upon strenuous muscle contractions, can damage the muscle cells via nicotinamide adenine dinucleotide phosphate oxidase-generated superoxide, thus resulting in lysis of the muscular cells^40^,^41^. The finding that muscle cell damage was not influenced by the infused H_2_-saline, however, suggests that the occurrence of muscular damages upon exercise in the present study was independent from the exercise-induced ROS generation, as indicated by the suppression of the elevated oxidative stress in H_2_-infused horses. Apart from chemical stress, including ROS, the muscle cell membranes are thought to be exposed to mechanical stress due to strenuous loading and intensive contraction of the muscle fibres during high-intensity exercise^14^. When mechanical stress overwhelms the muscular resistance, the sarcomere units, which are assembled from myofibrils and connective fibrils and fixed by structural proteins such as actin, dystrophin, and titin, become overstretched and disrupted, resulting in membrane failure and leakage of the sarcoplasmic components. In the present study, we observed a significant influx of CK into the serum after the race, regardless of the pre-treatment. In this case, the muscle damage seems to originate from mechanical stress; in spite of the increased oxidative stress and absence of responsive antioxidant potentials, as indicated by the serum 8-OHdG and BAP test results, the recovery from muscular damage, demonstrated by the CK values, was completed within 24 h after the race in both groups. In other words, the muscle damage seemed to be transient, suggesting prompt recovery of the muscle membrane and absence of ischemic tissue necrosis.

The resistance to the prolonged muscle damages observed in this study regardless of the administration of H_2_ could be explained by the adaptive response to the habitual endurance training of the horses. It has been demonstrated that endurance training induces mitochondrial biogenesis, represented by muscle fibre switching, which can improve energy metabolism and increase the resistance to fatigue as well as enhance the antioxidant potential^42^,^43^,^44^. It should be noted that the Thoroughbred horses included in the present study undergo daily training; this endurance training is thought to induce, at least in part, metabolic and antioxidant changes of the cellular defence mechanisms, which are believed to be modulated by PGC-1α^1^. It has been reported that PGC-1α is up-regulated in Thoroughbred horses undergoing high-intensity training for 18 weeks^45^. In addition, it is known that the muscles of Thoroughbred horses have a high proportion of type IIa fibres, which is consistent with the responsiveness to daily training^46^. In this study, the muscle fibres, particularly the type IIa fibres, may have been accustomed by the habitual endurance training, thus resulting in resistance to the increase of d-ROMs upon racing and in rapid improvement of the CK levels after the race.

Highly intensive exercises, which exhaust the anaerobic energy supply, require aerobic generation of ATP. Bioenergy in such strenuous exercises, including horse racing and human sprint athletics, is therefore provided by mixed and interlinked metabolic pathways composed of both anaerobic and aerobic reactions^47^. The present data obtained from Thoroughbred horses clearly demonstrated the influences of anaerobic metabolism as well as the presence of muscular damages. In the present study, a rapid and significant increase of serum lactate was observed (Table 1); the serum lactate levels peaked immediately after the race, clearly demonstrating the breakdown of free ATP and phosphocreatine, followed by the activation of anaerobic glycolysis. The notion of depletion of the anaerobic supply of ATP during racing is also consistent with the subsequent peak observed in the serum UA at 1 h after the race in both groups, which indicated insufficient re-phosphorylation of AMP during the race. Although a significant increase of UA immediately after the race compared to the baseline was observed only in the H_2_ group, no difference in the manner of energy supply was observed between the placebo and H_2_ groups. It should be noted here that, under such exhaustive circumstances, in which ischemia and hypoxia in the muscle are also induced, xanthine oxidase, which is converted from xanthine dehydrogenase, could generate superoxide via conversion of hypoxanthine to xanthine, and of xanthine to UA^48^. Further, apart from electron leakage through the mitochondrial membrane, another source of ROS may have appeared and contributed to the continuous oxidative state observed in the horses, which in turn may have been scavenged in the H_2_ group. The elevations of the UA and lactate levels in both groups are suggestive of post-exercise fatigue^8^,^13^. Although H_2_ may not improve such primary symptoms, the suppression of oxidative stress suggests a reduction of the secondary deleterious effects of racing exercise.

Finally, as mentioned, the horses used in the present study seemed to be equipped with adaptive antioxidant responses in the serum, as demonstrated by the suppression of the elevation of d-ROMs after high-intensity, exhaustive racing in both groups (Fig. 1b). The inveterate oxidative stress associated with high-intensity racing may induce late-onset or masked inflammation, which in turn is associated with increased risks of injuries, morbidity, and early mortality; this prolonged inflammation is accompanied and modulated by the elevated ROS via coupling with pro-inflammatory cytokines and NF-κB positive feedback loops^1^,^31^. Importantly, it should be noted that, unlike in the placebo group, significant suppression of the serum 8-OHdG was observed in the H_2_ group even 24 h after the race (Fig. 1a). This finding suggests that the administration of H_2_ may be applicable to many other situations in which oxidative stress is excessive, regardless of the status of the antioxidant defence. The possibility that oral daily consumption of water containing 5–7 ppm of H_2_ may counteract such oxidative risks has been hypothesised^31^,^49^. In addition, as demonstrated herein, it seems important to prepare for unexpected or dangerous oxidative stress increases that may overwhelm the effects of hydrogen-enriched water. Our findings suggest that one potential solution is infusion of H_2_-saline, and recent progress in the treatment of rheumatoid arthritis in humans using H_2_-saline supports this conclusion^50^.

There were some limitations to this study. Most importantly, collecting real-life data from habitually trained horses before and after racing under serious, strenuous conditions is an extensive task, and therefore, the number of horses studied was relatively small (N = 13) and a cross-over study design was not possible. However, although the present study was focused on the acute oxidative or antioxidative responses of Thoroughbred horses after racing, and although the data were restricted to within 24 h of the race, the remarkable reduction of serum 8-OHdG observed in the H_2_ group indicates a preventable potential of pre-treatment with H_2_-saline against the prolonged and detrimental effects resulting from exhaustive exercise. To confirm our findings, the effects of H_2_-saline injections on anaerobic metabolism should be further investigated, both in large-scale animal studies and in humans, in the future.

## Conclusions

Intravenous infusion of H_2_-saline significantly and integrally suppressed exercise-induced oxidative stress in Thoroughbred horses after exhaustive racing. Further, pre-exercise treatment also resulted in enhanced antioxidant potential. On the other hand, the transient muscle damages induced by the racing exercise, during which the anaerobic energy supply is depleted, were not affected by H_2_-saline injection. Taken together, the results of the present study clearly demonstrated that the direct injury caused by rapid and strong muscle contractions occurred independently from the generation of ROS. The antioxidant capabilities of H_2_ during exercise, in which inveterate oxidative stress significantly elevates the 8-OHdG levels, may aid the beneficial properties of exercise by enhancing the antioxidant potentials as well as the anti-inflammatory effects.

## Methods

### Subjects

Thirteen retired Thoroughbred racehorses, belonging to the Horseracing School of Japan Racing Association and regularly used by training jockeys, were randomly divided into a placebo group (N = 6; age: 4–11 years; 4 males, 2 females; body weight: 496 ± 35.1 kg) and H_2_ group (N = 7; age: 3–8 years; 4 males, 3 females; body weight: 487 ± 35.7 kg). There were no significant differences in the baseline characteristics between the two groups. All of the horses are trained 6 days a week, with each training sessions including 20 min of walking, 1,200 m of trotting, and 1,000 m of canter, followed by 2,000–3,000 m of gallop, and ending with cool down trotting and walking for at least 15 min.

### Preparation of H_2_-saline

To prepare the H_2_-saline, 1 L of saline in a soft plastic bag was placed for 12 h in a bath to circulate the saline. An electrolysis instrument (hydrogen circulator; Ecomo International Co. Ltd., Iizuka, Fukuoka-city, Japan) was used to generate 1.6 ppm H_2_. Two bags were placed in the infusion apparatus (INFUSTAT, Meiyu Co. Ltd., Yachio-city, Japan) and the H_2_ concentration was confirmed by using the methylene blue-platinum colloid regent-based titration method^51^. Just before the infusion, approximately 0.6 ppm of H_2_ was dissolved in the saline. Placebo saline was prepared in the same water bath without the addition of H_2_.

### Study design

This study was approved by and conducted in accordance with the guideline of the Animal Welfare and Experiment Management Committee of Miho Training Center, Japan Racing Association. The horses took part in a simulation race in which they ran 1,000 m in a dirt track. Immediately after removing the soft bags from the hydrogen circulator, 2 L of H_2_-saline was infused into the jugular vein of each horse over 5 min, 2 h before the race. Blood samples were collected from the jugular vein just before the infusion (baseline), and immediately (7–10 min), 1 h, 3 h, and 24 h after racing. All blood samples were put on ice immediately after collection, and the serum was separated by centrifugation and frozen at −80 °C. The blood lactate concentrations were analysed immediately after collection. The jockeys were not informed whether the infusion contained H_2_ or not, and were instructed to ride the horses as if they were taking part in a real, regular race.

### Biochemical analyses

To estimate the oxidative potentials in the serum samples, d-ROMs were measured as previously described^34^. This method is an indirect detection method of free radicals, including ROS, in which N,N-Diethyl-para-phenylenediamine reacting with hydrogen peroxide in the serum is measured by the change in the colour as the absorbance at 505 nm, using the Free Radical Elective Evaluator (WISMERLL Co. Ltd. Hongo, Tokyo, Japan). The results are presented as Carratelli units (U.CARR) based on the calculated absorption. Using the same apparatus, the BAP test was also performed to evaluate the reducing potential of the serum, in which the reduction of FeCl_3_ is detected as the disappearance of reddish colour^35^. Serum 8-OHdG, a marker for oxidative stress that reflects 8-hydroxyguanosine in the DNA, was measured using enzyme-linked immunosorbent assay (ELISA), as previously described^52^. The assay was performed using the highly sensitive ELISA kit for 8-OHdG (JaICA, NIKKEN SEIL Co., Ltd., Shizuoka, Japan). Serum CK, AST, LDH, and UA were measured at the Miho Training Center of the Japan Racing Association. Blood lactate was analysed using Lactate Pro2 (ARKRAY, Inc., Kyoto, Japan).

### Statistical Analysis

The data are shown as the mean ± standard deviation. Dunnett’s test was used for analysis of transition data using continuous measurements and for the baseline pre-dose absolute value in each group. For these analyses, *p* values < 0.05 were considered significant. Relative changes between the H_2_ and placebo groups were compared using Student’s t-test, with the level of significance set at *p* < 0.0125 after Bonferroni correction. All statistical analyses were performed using JMP v.6.0.3a software (SAS Institute, Cary, NC).

## Additional Information

**How to cite this article**: Yamazaki, M. *et al.* Intravenous infusion of H_2_-saline suppresses oxidative stress and elevates antioxidant potential in Thoroughbred horses after racing exercise. *Sci. Rep.* **5**, 15514; doi: 10.1038/srep15514 (2015).

## Figures and Tables

**Table 1:** Mean values of CK, AST, and LDH before and after racing.

	**Placebo (N = 6)**		**H2 (N = 7)**	
	**CK (U/L)**	***p*** **value**	**CK (U/L)**	***p*** **value**
Baseline	231 ± 74.0	—	186 ± 38.0	—
Immediately post-racing	290 ± 83.0	0.7186	252 ± 43.0	0.2802
1 h	306 ± 71.0	0.529	270 ± 78.0	0.124
3 h	385 ± 142	0. 0493*	354 ± 106	0.0006*
24 h	228 ± 77.0	>0.99	211 ± 48.0	0.916
	**AST (U/L)**	***p*** **value**	**AST (U/L)**	***p*** **value**
Baseline	318 ± 64.0	—	283 ± 45.0	—
Immediately post-racing	382 ± 62.0	0.2508	348 ± 70.0	0.1519
1 h	342 ± 31.0	0.9022	295 ± 58.0	0.9883
3 h	369 ± 63.0	0.435	330 ± 59.0	0.4066
24 h	339 ± 58.0	0.9372	295 ± 36.0	0.9888
	**LDH (U/L)**	***p*** **value**	**LDH (U/L)**	***p*** **value**
Baseline	478 ± 118	—	417 ± 100	—
Immediately post-racing	569 ± 125	0.5782	500 ± 96.0	0.386
1 h	555 ± 74.0	0.7042	449 ± 102	0.9422
3 h	523 ± 138	0.9352	450 ± 116	0.9331
24 h	397 ± 134	0.6669	345 ± 48.0	0.493

Abbreviations: AST, aspartate aminotransferase; CK, creatine kinase; LDH, lactate dehydrogenase.

Baseline indicates the values just prior to injection.

Values are presented as mean ± standard deviation.

^*^Significant increase vs. baseline.

**Table 2:** Mean values of serum lactate and UA before and after racing.

	**Placebo (N = 6)**		**H2 (N = 7)**	
	**Lactate (mmol/L)**	***p*** **value**	**Lactate (mmol/L)**	***p*** **value**
Baseline	0.52 ± 0.16	—	0.43 ± 0.21	—
Immediately post-racing	15.0 ± 2.22	<0.0001*	16.4 ± 2.61	<0.0001*
1 h	7.38 ± 1.27	<0.0001*	7.89 ± 2.97	<0.0001*
3 h	0.88 ± 0.12	0.942	0.84 ± 0.20	0.9742
	**UA (mg/dL)**	***p*** **value**	**UA (mg/dL)**	***p*** **value**
Baseline	0.17 ± 0.05	—	0.20 ± 0.06	—
Immediately post-racing	1.10 ± 0.29	0.1151	1.06 ± 0.36	0.0095*
1 h	3.50 ± 1.42	<0.0001*	3.53 ± 0.86	<0.0001*
3 h	0.83 ± 0.29	0.3476	0.81 ± 0.39	0.0872
24 h	0.13 ± 0.01	0.999	0.14 ± 0.05	0.9983

Abbreviations: UA, uric acid.

Baseline indicates the values just prior to injection.

Values are presented as mean ± standard deviation.

^*^Significant increase vs. baseline.
